# Structured sequence processing and combinatorial binding: neurobiologically and computationally informed hypotheses

**DOI:** 10.1098/rstb.2019.0304

**Published:** 2019-12-16

**Authors:** Ryan Calmus, Benjamin Wilson, Yukiko Kikuchi, Christopher I. Petkov

**Affiliations:** Newcastle University Medical School, Framlington Place, Newcastle upon Tyne, UK

**Keywords:** computational modelling, sequence learning, serial order, chunking, language, binding

## Abstract

Understanding how the brain forms representations of structured information distributed in time is a challenging endeavour for the neuroscientific community, requiring computationally and neurobiologically informed approaches. The neural mechanisms for segmenting continuous streams of sensory input and establishing representations of dependencies remain largely unknown, as do the transformations and computations occurring between the brain regions involved in these aspects of sequence processing. We propose a blueprint for a neurobiologically informed and informing computational model of sequence processing (entitled: Vector-symbolic Sequencing of Binding INstantiating Dependencies, or VS-BIND). This model is designed to support the transformation of serially ordered elements in sensory sequences into structured representations of bound dependencies, readily operates on multiple timescales, and encodes or decodes sequences with respect to chunked items wherever dependencies occur in time. The model integrates established vector symbolic additive and conjunctive binding operators with neurobiologically plausible oscillatory dynamics, and is compatible with modern spiking neural network simulation methods. We show that the model is capable of simulating previous findings from structured sequence processing tasks that engage fronto-temporal regions, specifying mechanistic roles for regions such as prefrontal areas 44/45 and the frontal operculum during interactions with sensory representations in temporal cortex. Finally, we are able to make predictions based on the configuration of the model alone that underscore the importance of serial position information, which requires input from time-sensitive cells, known to reside in the hippocampus and dorsolateral prefrontal cortex.

This article is part of the theme issue ‘Towards mechanistic models of meaning composition’.

## 1. Introduction

Natural environments are richly structured in both space and time. Substantial progress has been made in understanding the neurobiological bases of learned relationships between spatially or temporally separated elements [1–3]. Moreover, prior research has established the importance of serial order for the brain [4], and *binding problems*, whereby distinct sensory events are combined for perception, decision and action [5], have attracted considerable interest and empirical enquiry [6,7].

Establishing relationships, or *dependencies*, between elements over time allows us to extract the structure of the sensory world and to make predictions about future events. However, understanding how the brain binds complex information distributed in time, building temporally organized structures that represent multiple linked dependencies, remains a considerable challenge facing the neuroscientific community. This sort of cognitive structure-building is challenging for the brain to achieve because complex input must be discretized in time, resultant discrete items chunked and stored in memory, dependencies identified and related items bound for perception (or other purposes), and representations of multiple dependencies maintained concurrently in memory to be further manipulated [8,9].

Human language—written, spoken or signed—is a salient example of the complexity of the binding problem, because it features syntactically organized dependencies between semantic units [10]. Yet the problem of building complex representations is also relevant to complex action sequences [8,11,12], music [8,13,14], mathematics [9,15] and cognition in general [11,13]. Moreover, some of these systems are not unique to humans, since songbirds can construct complex vocalization sequences [16], an ability supported by a forebrain neural system [17], and correspondences have been established between humans and a number of species in processing adjacent and non-adjacent sequencing dependencies [18–20]. Thus, advancing our understanding of how complex structure can be built from sequential input in computational and neural systems is important for developing better machine and animal models to understand both general principles and species-specific aspects of combinatorial binding.

In this article, we propose a blueprint for a neurobiologically informed and informing computational model of sequence processing (entitled: Vector-symbolic Sequencing of Binding INstantiating Dependencies, or VS-BIND). The VS-BIND approach integrates: (1) advances in modelling combinatorial binding within simulated neural systems using vector symbolic operations; (2) insights from neuroimaging and neurophysiological evidence in human and non-human primates on neural correlates of structured sequence processing and working memory; and (3) dynamic mechanisms for manipulating population codes that can be incorporated in modern spiking neural networks [21,22]. The approach allows us to plausibly transform internal representations, rendering these into both mathematically idealized and neurally simulated site-specific activity unfolding over time. Building on these foundational mechanisms, we focus on modelling chunk encoding and the binding of sensory items to represent adjacent, non-adjacent and more complex (hierarchically) structured sequencing dependencies. Our key objectives here are to motivate the approach, ground it in the relevant literature, and use it to generate distinct mechanistic predictions, the form of which, as will be seen below, depends on both the specific binding operations used and their configuration.

It is important to note that any model using combinatorial operators can only be described as *classically compositional* if combinatorial representations precisely reflect the meaning of all constituents and the relations between them. The operators used here are not classically compositional, but nevertheless serve an important purpose in allowing us to generate falsifiable predictions of neural mechanisms and correlates of structured sequence processing ripe for neurobiological testing across the species. To assist in this process, we also share Matlab (MathWorks) code, including a demonstration (doi:10.5281/zenodo.3464607) [23]. Even on the basis of its structure alone and initial modelling, the VS-BIND approach posits a number of intriguing predictions.

## 2. Foundations of descriptive and computational models of structured sequence processing

Language relies on semantic and syntactic knowledge, supported by the detection of dependencies between phonemes, morphemes, words and phrases in sentences. The language binding problem features the rapid detection of lexical symbols and the encoding of complex syntactic regularities at multiple scales and temporal granularities.

A large volume of neurobiological data implicate a fronto-temporal brain system in various aspects of language processing [11,24,25]. Neurobiological signals associated with the chunking and parsing of speech with respect to phrase boundaries have been identified in these regions [26–28]. Moreover, the temporal structure of speech or language content at different timescales (phonemic, syllabic, word or phrasal) produces stimulus- or context-driven neural entrainment at the relevant oscillatory frequency bands [29,30].

Behaviourally, a number of sequencing processes are now known to be evolutionarily conserved, including entrainment to rhythmic sensory input [29,31]. There is also information from artificial grammar learning paradigms, which are used to establish dependencies between otherwise arbitrary auditory or visual items in a sequence, either via statistical or rule learning [32,33]. Humans and a number of non-human animals can learn dependencies between sensory items next to each other in a sequence (*adjacent dependencies*), as well as dependencies further separated in time and by intervening items (*non-adjacent dependencies*; reviewed in [18]). The learning of hierarchically organized dependencies by non-human animals is, however, contentious and it remains to be seen whether this ability is uniquely human [34,35].

**Figure 1.**
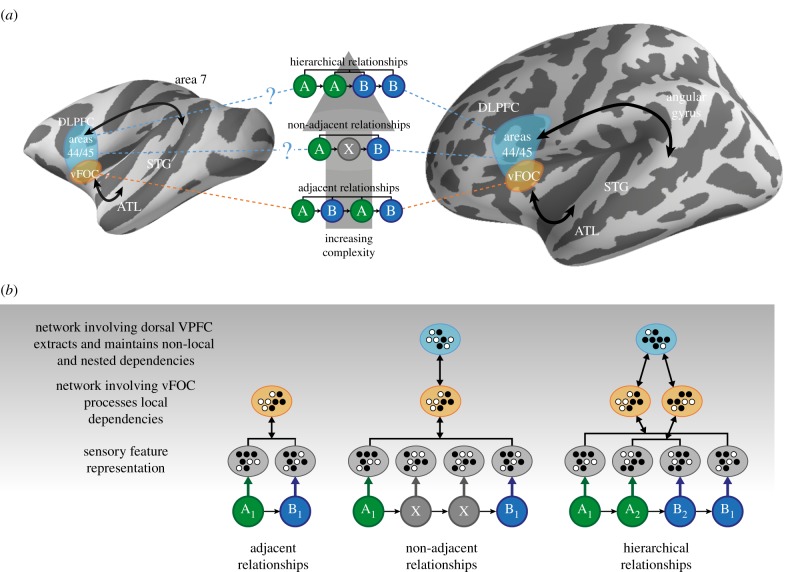
Neurobiologically informed heuristic model of structured sequence processing, by Wilson, Marslen-Wilson and Petkov. (*a*) Fronto-temporal regions involved in sequence processing, from [34]. DLPFC, dorsolateral prefrontal cortex; vFOC ventral frontal opercular cortex; ATL, anterior temporal lobe; STG, superior temporal gyrus. (Copyright © 2017 Benjamin Wilson, William D. Marslen-Wilson and Christopher I. Petkov, CC BY 4.0.) (*b*) Predicted combinatorial codes illustrated as neural patterns implemented by coordination between different regions. (Online version in colour.)

Comparative neuroimaging work has identified brain regions in human and monkey frontal and temporal cortex involved in processing sequencing dependencies [19,20]. This has led to descriptive models of the brain-bases of structured sequence processing and the relationship with, and distinctions from, neurobiological processes involved in language [34,36,37]. For the purposes of this paper, we will focus on the Wilson *et al*. [34] descriptive neurobiological model of human and non-human primate structured sequence processing, shown in figure 1. Relatively simple sequencing relationships, such as between items that occur next to each other in a sequence, can be learned by both humans and monkeys and are seen to engage corresponding brain regions, particularly the ventral frontal and opercular cortex (figure 1*a*, vFOC, bottom row) [34]. For non-adjacent dependencies (which increase working memory demands: store an item in memory long enough to link to its matched pair), it is less clear whether the frontal operculum, other inferior frontal areas (such as areas 44/45), and/or the dorsolateral prefrontal cortex (DLPFC) are more involved. More complex (including hierarchical) dependencies engage inferior frontal areas 44/45 in humans (Broca's area; see figure 1*a*, middle and top rows) [38]. The hippocampus has also been implicated in structured sequence processing [39,40] and implicit learning [41], but its mechanistic role within these contexts remains incompletely understood.

Computational models of language or the processing of serial information provide compelling simulations of behavioural data [42,43]. However, modelling underlying neural mechanisms presents additional challenges. David Marr's tri-level framework [44] famously defines three levels of description that are still widely applied in characterizing any given model of the brain: the goals of the system (*computational* level), the cognitive processes required to reach this goal (*algorithmic* level), and the neural mechanisms required to instantiate them (*implementational* level). Poggio [45] extended this framework by suggesting that models should also offer insights into learning processes and the evolutionary path that yields the system [46]. Although advances have been made in understanding many neuropsychological phenomena at individual levels of description, it remains desirable to advance understanding on multiple levels through holistic modelling approaches [47].

The fields of neuroscience and machine learning have been converging, in particular through the use of models incorporating functionally and anatomically distinct subpopulations of artificial neurons [48]. Artificial neural networks (ANNs), including deep and recurrent neural networks (DNNs and RNNs), are the dominant connectionist modelling paradigm in use today, using iterative training procedures to tune synaptic weights between artificial neurons and establish network-level computations. RNNs are relevant to sequence learning since recurrent feedback allows them to integrate information over time [49], while DNNs have revolutionized machine learning, increasingly inform neuroscientific analyses, and can generate neural correlates [50].

A likewise informative, but paradigmatically distinct approach is to model the brain at an algorithmic level, explaining behaviour and cognition in terms of computational processes that combine and transform cognitive symbols (see [51]). Reconciling neural and cognitive perspectives is a longstanding challenge, but there exists a computational modelling subfield that has made considerable strides in this direction: *symbolic connectionism*. This approach seeks to produce ANN models with explicit support for combinatorial and symbolic operations (*neural–symbolic* networks). This is the subfield we look to in furthering our modelling aims, specifically using *vector symbolic architectures* (VSAs; see below), which can be used alone, in ANNs, or in dynamic spiking neural networks. Our use of VSAs has the benefit of generating predictions on neural mechanisms of combinatorial binding throughout the fronto-temporal system involved in structured sequence processing.

## 3. Computationally modelling structured representations in neural systems: an overview of approaches

There exist a number of symbolic connectionist solutions, and many non-symbolic or non-connectionist cognitive architectures, each addressing various aspects of neural binding. Here, we briefly overview symbolic connectionist approaches before justifying our use of VSAs. For brevity, we restrict discussion here only to approaches that specify ways to build structured representations while explicitly supporting neurobiologically plausible implementation.

Three distinct methods of modelling binding predominate, although they can in many ways be viewed as complementary [52]. The first uses coordinated temporal synchrony (see LISA [53,54] and SHRUTI [55]) or asynchrony (see DORA [56]) to unify constituents. The second uses uniform grids of integrating circuits to create a stable memory for bindings (neural blackboard architectures, NBAs [57]). The third, encompassing VSAs, principally uses conjunctive spatial coding of abstract vector representations to associate items, via tensor products [58–60], circular convolution [61] or other defined transforms [62,63].

Although commonly contrasted, spatial and temporal binding mechanisms are not mutually exclusive. Conjunctive spatial coding and temporal synchrony/asynchrony are known to be complementary and can be thought of as different perspectives on the same dynamic process [64]. Furthermore, conjunctive coding is considered appropriate for long-term storage in temporally coordinating models [52,65], whilst vector-based methods can operate within dynamic frameworks likewise subject to temporal influences [22], which we will consider in more detail later. Thus, spatial and temporal binding approaches are not diametrically opposed but rather place different emphases and explanatory burdens on mutually informing aspects of neural coding. Although VSAs tend to be conceived of as static systems, we specifically advocate their use within a dynamic framework (for example, Nengo [21,22]) to incorporate advantages of both temporal and population coding.

The neural population codes employed by these models vary. In this regard, *localist* representations (which exhibit one-to-one or many-to-one mappings between features and neural activation) are typically contrasted with *distributed* representations (which exhibit many-to-many mappings). Distributed representations may be *dense* or *sparse*, which in the latter case means that a low proportion of neurons are active within a population at any one time. There is evidence that both localist [66,67] and sparse distributed representations are used by the brain [68–70], and there are advantages to both encoding strategies for neural systems. For instance, localist representations exhibit the lowest possible interference between encodings, whilst sparse distributed representations exhibit graceful degradation in performance in the presence of increasing noise [71]. Sparse distributed vectors exist on a representational continuum that allows them to demonstrate characteristics of either localist or distributed encodings depending on their sparsity [72]. This flexibility motivates the use of abstract vector systems that define combinatorial operators over sparse distributed representations. VSAs [73], which we use to implement VS-BIND, accomplish this.

These models also differ in terms of how semantic representations are instantiated [74]. Some define the semantic structure of relational encodings at the neural level, generating explicit *role–filler* [53,55] or *symbol–argument* bindings [59]. These models provide clear mechanisms to support compositional relational encodings of semantic knowledge, where the whole perfectly reflects its parts, a feature considered important for linguistic modelling [53–56]. By comparison, VSAs do not inherently specify neural implementations at all, but are rather supported in this regard by broad theoretical frameworks that specify mappings between abstract vectors and neural population codes (for example, the semantic pointer architecture [22], used in Nengo [21], or integrated connectionist/symbolic architecture, ICS [75]).

*Structured sequence processing* typically focuses on ordering relationships and can refer to operations on meaningless items (e.g. nonsense words or abstract visual shapes). We consider cognitive architectures incorporating VSAs [58,61–63] to have particular strengths appropriate to their use in a neurobiologically plausible model of structured sequence processing. Firstly, using existing tools, VSAs can act as a bridge to multiple modelling paradigms. For example, Nengo, a library for large-scale dynamic neural simulation [21], supports VSA encodings within spiking neural networks through the use of the Semantic Pointer Architecture. Bayesian computations [76] and attractor dynamics [77] have also been instantiated within this spiking neural network system. Secondly, VSAs are highly scalable solutions possessing substantial storage capacity for high-dimensional information [78]. Thirdly, as we describe shortly, VSAs permit the definition of relationships at the algorithmic level using an easily interpretable algebra. Finally, VSAs remain neurocomputationally plausible [22] whilst being relatively straightforward to implement [61,62,79]. To describe our approach, we next outline VSA principles and operators, before explaining how temporal dynamics can control such operations. We conclude by describing the role of these mechanisms within a neurobiologically plausible model of structured sequence processing, VS-BIND.

## 4. Combinatorial population coding with vector symbolic architectures

**Figure 2.**
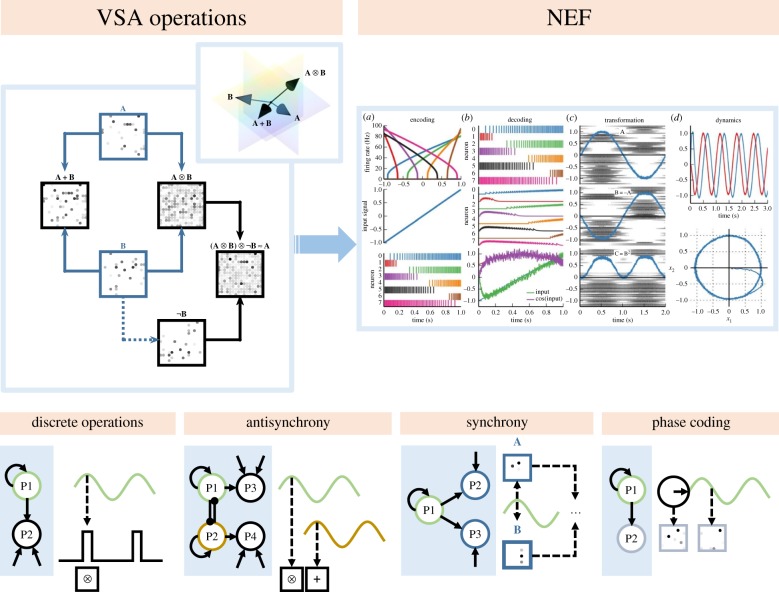
Spatial and temporal coding within a spiking model. *Top row, left panel*: vector symbolic architecture (VSA) operations using circular convolution to accomplish binding (**A** ⨂ **B**) [61]. Sparse, high-dimensional random vectors represent distinct symbols **A** and **B** (blue text). To aid visual comparison, vectors are shown reshaped into squares, meaning the dimensionality of each vector equals the number of ‘pixels’ in each box (here, 256-dimensional, plotted as 16 × 16). These vectors can also be considered *directions* in high-dimensional space (inset, projected down to 3D using principal components analysis). Results of the VSA operators are shown (upper left main panel, black text, clockwise from left: *superposition*, *binding*, *unbinding* and *inversion*; see text, §4), with arrows indicating the flow of operands. A noisy recovered vector (rightmost square) can be cleaned up with an autoassociative memory to produce **A** (top square). *Top row, right panel* (from [21]; copyright © 2014 Bekolay, Bergstra, Hunsberger, DeWolf, Stewart, Rasmussen, Choo, Voelker and Eliasmith, CC BY 3.0): Core properties of the Neural Engineering Framework (NEF) [22] as implemented in Nengo [21]. To the left are tuning curves of individual neurons (*a*, top plot). In vector terms, each neuron fires maximally to its own *preferred direction*. Nonlinear *encoding* of an input signal (*a*, middle plot) yields spike trains for each neuron (*a*, bottom plot). *Decoding* (*b*) is possible using linear methods. Combining decoding with encoding, one can determine synaptic weights representing transformations between populations (*c*). Here, VSA representations are simply high-dimensional signals encoded like any other. Operations like convolution can be learned by simulated spiking networks incorporating spike-timing-dependent plasticity (STDP) in Nengo [22]. Finally, dynamic signals can be represented (*d*), of relevance for understanding oscillatory mechanisms [26,28,29]. Nengo is agnostic about neural models, with many spiking models available [80–82]. *Bottom row:* since the NEF provides mechanisms for spatial (VSA operations) and temporal (dynamic) manipulation of representations, possibilities exceed that of a static system. Simple interactions between segregated populations (*networks shown in blue boxes*) lead to controlled functional relationships. Thresholded dynamic activity, e.g. arising from an oscillator (P1, *leftmost panel*) can trigger discrete combinatorial operations. These operations can be segregated over time (*middle left panel*) through control by interacting, antisynchronous oscillations (P1 and P2). Likewise, common driving signals can synchronously strengthen representations in disparate regions (*middle right panel*, P1 multiplicatively modulating P2 and P3) for downstream processing such as feature binding. Finally, the *phase* of an oscillator, rather than its amplitude (*rightmost panel*) can drive downstream encodings such as those of relative position. (Online version in colour.)

In VSA models, the basic units of representation are high-dimensional vectors. These typically sparse representations (figure 2, vectors **A** and **B**) can be visualized directly (figure 2, top left panel) or encoded as neural activity using multidimensional tuning functions (figure 2, top right panel) [21,83].

Symbolic vectors can be recombined using specific, reversible combinatorial operators to create new representations containing information on their constituents and the relations between them [58,61,62]. Inputs to these operations can either be atomic vectors (those that are not compound representations) or the results of previous operations. Atomic vectors are often randomly generated in VSA models, but can also be generated by compressing even higher dimensional input. Using this latter approach, semantically similar concepts cluster together in vector space [22].

The power of VSA approaches lies in their combinatorial operators. Although these are broadly common to all VSAs, here we use only one VSA, that of Plate [61]. This VSA uses the following operators: *superposition* (or *bundling*; point-wise vector addition of inputs), *binding* (a conjunctive operator) and its inverse, *unbinding* (also termed *release*, which here relies on *inversion*, equivalent to logical ‘not’), and a vector *comparison* operator for readout of results. Note that within VSA terminology, only the conjunctive operation is known as *binding*, but both this specific operator and superposition fit the wider definition of binding in the broad context of neural binding problems [5]. The more specific VSA nomenclature, which we adhere to hereafter, is helpful because it imposes constraints on the potential neural mechanisms involved in each of these combinatorial processes.

Using the full set of VSA operators, algorithmic manipulations are undertaken easily. The operators are best demonstrated with just two inputs (here **A** and **B**; typical VSA operators are illustrated in the top left panel of figure 2). **A + B** (*superposition*) yields a vector *correlated* with both **A** and **B**. This is simple vector addition, essentially overlaying the sparse inputs. **A** ⨂ **B** (*binding*) is a conjunctive operator that yields a vector approximately *orthogonal* to both A and B, and as such is poorly correlated with either input. In this VSA, binding is calculated through circular convolution [61], which is just one possible way to create an output vector of the same length as one input alone [62,63]. This feat is possible because the operator encodes a *reduced representation* of both inputs, which can be unbound as described below.

Reduced representations are an important feature of a number of VSAs. Without them, conjunctive operations on input vectors of length *N* each result in output vectors of length *N*^2^ (leading to exponential increases in vector size, a *combinatorial explosion*). This is a characteristic of the ancestral VSA, Smolensky's *tensor product binding* model [58,75]. Plate's [61] VSA, by contrast, overcomes this scaling problem by making use of compressive representations known as *holographic reduced representations* (HRRs). Reduced representations allow a single model to support repeated (or recursive) operations on vectors without dimensionality increasing; the limiting factor in using reduced representations is instead that the process is lossy, so repeat operations cumulatively degrade the output vector. It is partly for this reason that this approach cannot be considered classically symbolic or perfectly compositional. However, lossy encoding recapitulates natural limits on working memory and the depth of recursively nested structures that can be constructed or comprehended in natural language [84].

Here*, unbinding* is in essence binding, but with a change to one of the operands. Values may be unbound or *released* from a bound representation by computing a new binding between it and the *approximate inverse* of one of the original inputs with respect to the binding operator (from hereon in, just named the *inverse*, ‘¬’; see **¬B,** figure 2). Inversion is accomplished by simply permuting all but the first dimension of **B**, which at a neural level means rerouting input dimensions using a distinct pattern of synaptic weights. It is easier to understand the inversion operator by demonstrating its use in unbinding; the vector symbolic formula **(A** ⨂ **B)** ⨂ **¬B** shown in figure 2 depicts this process. Here, we bind the bracketed representation with a ‘key’ containing the inverse of the element we know to be linked to the vector we wish to recover. Without such a key it is impossible to retrieve the contents of a binding via circular convolution. This means that VSA operations must be configured carefully to ensure that bound information can be retrieved in a plausible way.

To interrogate the result of unbinding, or any VSA operation, we can use the comparison operator to assess the similarity of the output to a defined vocabulary of representations. This can be useful as part of an autoassociative memory, or to control downstream actions or operations. For example, let **R** = **(A** ⨂ **B)** ⨂ **¬B**. Here, undertaking a comparison between **R** and a set of two vectors {**A**, **B**} would reveal that **R** is highly similar to **A** and highly dissimilar to **B**. Comparisons can be conducted on real-valued vectors using correlation or the vector dot product (where a smaller angle between input vectors indicates stronger similarity). The dot product in particular could be implemented by any neuron; it is equivalent to simply summing all presynaptic potentials weighted by their respective synaptic weights over a short period of time [70].

Use of VSA operations is by no means restricted to elementary computations. Mathematically, the superposition (+) and binding (⨂) operators are designed to exhibit associativity, commutativity and distributivity akin to their scalar analogues, addition and multiplication [61]. For example, if **A**, **B** and **C** each represent vectors, the vector symbolic formula **A** ⨂ (**B** + **C**) is equivalent to (**A** ⨂ **B**) + (**A** ⨂ **C**), just as one would expect algebraically. This makes the results of multiple VSA operations predictable and transparently interpretable. In a similar fashion, unbinding still functions if we superpose multiple bindings, as in **R** = (**A** ⨂ **B**) + (**C** ⨂ **D**). In this case, **R** ⨂ ¬**D** will result in the value (**C** + *noise*), recovering a representation highly similar to **C**.

Are such operations neurobiologically plausible? Plausibility of the neuronal arithmetic at a basic level is well supported; additive and multiplicative functions, which are sufficient to compute all of the VSA operations described here, including circular convolution, are established neural processes [85]. Multiplicative and divisive functions abound in the cognitive neurobiological literature, as feedback influences on neural responses [86,87]. The circular convolution operator could also be substituted for a number of alternative conjunctive distributed operators, for example, vector-derived transformation binding [63]. There is a good deal of evidence consistent with the presence of conjunctive distributed encodings in associative areas such as retrosplenial cortex [88], the ventral visual stream [89], and hippocampal CA1 and CA3 subregions [90].

How do we distinguish superposition from multiplicative [72] operations like convolutional binding in high dimensions? We expect that neurons instantiating additive operations should demonstrate linear responses to linear combinations of features of constituent representations, whilst multiplicative operations should result in nonlinearly selective responses. Empirical evidence already suggests that superposition cannot be the only combinatorial function, since linear and nonlinear mixed selectivity are *both* prevalent within the lateral prefrontal cortex and elsewhere [91,92]. Thus, there is evidence for the broad classes of functions to which the VSA operators belong in regions supporting cognitive function.

Simulations likewise demonstrate plausibility; Plate's VSA operators, discussed here and shown in figure 2 [61], form the combinatorial backbone of Eliasmith's Semantic Pointer Architecture, a neurobiologically plausible representational framework underlying Nengo, a Python library supporting the construction of spiking neural models [21,22]. The SPA proposes that high dimensional representational vectors arising in sensory cortex are compressed by similarity-preserving dimensionality reduction, and that the brain undertakes vector symbolic operations on these reduced representations. Nengo supports the transcoding of these cognitive representations into spiking neural representations by applying a set of fundamental neural encoding and decoding principles, the Neural Engineering Framework (NEF) [21], summarized with reference to the original paper in figure 2 (upper right panel and figure legend). Under this system, each spiking neuron contributes to a distributed encoding of an underlying latent vector representation; that is, there is a many-to-many mapping between the dimensions of a cognitive vector (itself already a distributed representation) and individual neurons. The resulting spiking activity is inherently dynamic, and thus suitable for manipulation over time by temporal mechanisms within the same dynamic framework.

## 5. Dynamically coordinating combinatorial operations with temporal mechanisms

As we have seen, population coding and temporal mechanisms are both functionally important to any account of domain-general structure-building [26,72]. Here, we introduce basic oscillatory principles that serve as temporal mechanisms within VS-BIND.

Neural oscillations in the brain reflect temporally coordinated responses of neural populations [93,94]. Oscillatory signals can also result in oscillatory *coupling* across frequency bands that reflects coordinated interactions within and between brain regions [94,95]. Theta-gamma coupling, for example, is associated with cognitive function and is also seen during structured sequence processing tasks [94,96].

We can instantiate dynamic relationships, including coupling, within a spiking neural model such that they undertake functionally useful coordinating or multiplexing roles in neurobiologically plausible ways (figure 2, bottom row). For example, a self-connected population (leftmost panel, P1) can generate oscillatory dynamics that, when thresholded, serve as the trigger for discrete vector symbolic operations on multiple inputs (P2). Likewise, interacting oscillators (P1 and P2, middle left) can exhibit antisynchrony, segregating discrete operations such that they do not interfere. Temporal synchrony can also be simulated (middle right) through top-down influences on multiple neural populations, for example by controlling gain, such that downstream operators (for example, VSA operators) act on coordinated inputs. Here, temporal coordination serves to functionally associate two vector representations that would otherwise remain separate.

The dynamic context in which these spatial operators act is crucial, because it serves to mitigate the concern that, owing to their multiplicative interactions, such spatial bindings are too variable to support generalization over classes of their inputs, and thus insufficient as relational encodings, a problem characterized in the literature as *violation of role–filler independence* [72]. The explicit segregation of representations in space (figure 2, **A** versus **B**) and time (figure 2, top left panel, upstream/downstream) means that multiple neural ensembles concurrently instantiate different components of a combinatorial representation. Variability in the downstream binding of **A** and **B** would not prevent either of these two populations from generalizing over their inputs. Constituents may be dynamically bound or unbound as needed to segregate or aggregate information.

Finally, oscillatory signals can unidirectionally coordinate activity elsewhere, for example on the basis of phase (figure 2, bottom row, rightmost panel), thus potentially exhibiting phase–amplitude coupling effects. This produces behaviour consistent with conceptual models on the role of theta–gamma coupling [97] observed in the brain [94,98]. VS-BIND exhibits phase–amplitude coupling as an explicit functional property of relative position coding.

## 6. Network-level mechanistic hypotheses derived from VS-BIND

Our objective within the remainder of this paper is to outline the specific combinations of operations required to support dependency encoding during sequence processing, generating neurobiological hypotheses for adjacent, non-adjacent and hierarchical dependency encoding. The Matlab demo provided at doi:10.5281/zenodo.3464607 further illustrates key principles outlined here.

### (a) Adjacent dependencies

We first consider the binding of two items following each other closely in time. At the vector symbolic level, ordered sequences can be unambiguously represented using one of two principal methods. The first is by encoding elements with respect to each other (*chaining*). However, behavioural findings in multiple species do not convincingly support this approach [99]. An alternative method, encoding each element with respect to a serial *positional tag* (*Sequence* *=* 1^st^ * Item1 *+* 2^nd^ * Item2, and so on), has found greater support. Behavioural results in songbirds suggest a reliance on positional cues during sequence recognition [100]. Likewise, during recall, humans and non-human primates are more likely to confuse items in different memorized sequences if those items share the same ordinal position across sequences [9]. Positional influences on recall provide behavioural evidence that ordinality is incorporated into the encoding of sequences even when position is not explicitly featured in a task, offering support for positional tagging.

**Figure 3.**
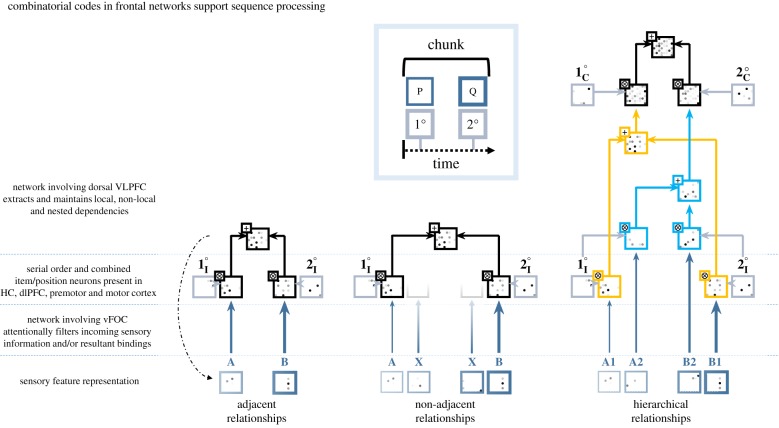
Neurobiologically informed vector symbolic encoding of sequence structure. Vector symbolic operators can account for the processing of a variety of sequencing dependencies. The solid arrows in these charts indicate the flow of information during *encoding* of a stimulus sequence only. These describe transformations of latent vector symbolic representations, as opposed to neural activation patterns. Representation strength is denoted by the shading and thickness of each box border. For clarity, representations are shown separated along the horizontal axis, though separate boxes do not necessarily imply separate neural populations are engaged, especially if describing identical computations, which could be undertaken by neurons of a single region. We suggest sensory representations (bottom row) are maintained within the supplementary motor area (not shown) and retrieved as needed. Operations unfold dynamically following principles outlined in figure 2. The final encoded sequence representation is found at the top of each diagram. Each is a reduced representation whose constituents can be inspected without serially unpacking all bindings; the superposed final result of the *adjacent relationship* encoding (leftmost diagram), for example, can be interrogated to recover its secondary element by simply binding it with ¬**2**°_**I**_. Serial elements packaged into a single representation are considered to be *chunked* in the traditional sense (inset box), but identical operations can be applied to non-adjacent (middle diagram) or nested pairs of elements (rightmost diagram), using separate item (I) and chunk (C) position encodings. Selective fading along the vertical axis represents salience filtering in the non-adjacent example (middle). Finally, the dashed, curved arrow shows just one case in which *sub-symbolic* feedback from a cognitively abstract cortical region might ultimately influence the representation of individual elements in sensory cortex (there may be many such pathways, but one exemplar is shown). Thus, although the figure, for simplicity, suggests VS-BIND is largely a feedforward model, feedback influences feature and can, for example, allow certain areas to influence sensory cortical representations. (Online version in colour.)

Detection of ordinal serial position, an essential component of sequence encodings within VS-BIND, we posit involves DLPFC, and motor and premotor cortex. Electrophysiological findings in non-human primates align with this account, revealing populations of cells in each of these regions involved in consistently encoding serial position irrespective of stimulus identity [101,102]. Studies of the hippocampus likewise reveal temporal coding relative to stimulus presentation, hypothesized to form part of the context for later retrieval [103]. There is thus a neurobiological basis for the crucial role played by explicit positional tags within our model (figure 3, light blue–grey boxes). Within VS-BIND, positional tags are considered to be deterministically but flexibly generated by the brain.

Here, tags follow the nomenclature ‘primary’ and ‘secondary’ (1° and 2°) rather than the ordinal absolutes ‘first’ or ‘second’, in line with human and non-human primate behavioural [104–106] and electrophysiological [101,103] evidence suggesting that, within sequences of words or actions, ordinal position is encoded relative to sequence boundaries rather than in absolute terms. Within our model, positional tags are anchored to the boundaries of perceptual chunks (figure 3, box inset) by decoding stimulus-entrained oscillatory phase (figure 2) such that both the sequence items and their relative positional tags are derived from the sensory input. It is important that each tag remains orthogonal to the last, which is a requirement for later unambiguous recovery of specific elements in a sequence.

To encode a sequence, continuous input over time is first discretized. These discrete sensory items are bound to distinct positional tags (to form position–item representations) and superposed in a decaying, recurrently connected working memory buffer. This *sequence buffer*, likely supported by SMA and pre-SMA [107], therefore maintains a linear, ordered representation of the input sequence (i.e. *Sequence* *=* *1°* ⨂ *Item1* *+* *2°* ⨂ *Item2* *+* *3°* ⨂ *Item3*). From this, individual sensory representations can subsequently be retrieved (via *Item* ≈ *Sequence* ⨂ ¬*Position*) and recoded to reflect dependencies between items or chunks (figure 3, bottom row, showing retrieved items). During this recoding process, maintained representations (**A**, **B**, or irrelevant intervening items, **X**) serially accumulate within a *dependency buffer* over time (i.e. moving rightwards) where they can be used in increasingly complex binding operations (moving upwards). The SMA encoding steps are comparable to the VSA approach in the ordinal serial encoding (OSE) working memory model of Choo & Eliasmith [99], which is capable of modelling behavioural characteristics of serial recall such as working memory *primacy* and *recency* effects. However, unlike the OSE model, VS-BIND incorporates centrally coordinating oscillatory activity and uses boundary-relative (rather than absolute) ordinal codes. Moreover, subsequent to serial encoding within working memory, VS-BIND describes the encoding of various dependencies, which need not be linear in organization (figure 3).

To encode dependencies, items are retrieved from sequence memory and bound with new positional tags (figure 3, light blue–grey boxes**)**. As in sequence memory, bindings form over time between tags and corresponding items, which are superposed to form representations of specific dependencies (figure 3, topmost representation, all diagrams). The timing of each binding operation is associated with and coordinated by stimulus-related oscillatory activity (figure 2) [29,31].

### (b) Non-adjacent dependencies

To support efficient encoding, and permit generalization from learned adjacent dependencies, simple non-adjacent relationships may be encoded in an equivalent manner to adjacent dependencies. To accomplish this, all constituent items must be maintained in the sequence buffer long enough to be integrated, and maintained representations need to be selectively propagated to the dependency buffer. This process involves salience filtering (e.g. by repetition suppression, expectancy or attentional processes [108]; figure 3, centre diagram), possibly supported by regions such as ventral frontal cortex including the frontal operculum. This is based on findings that the frontal operculum appears to be more active during the presentation of infrequent or novel auditory cues [109] and responds preferentially to violations of adjacent dependencies [38]. We suggest that this region integrates information from working memory as soon as it is available, but only maintains it over a relatively short time period. In this case, preferential responses to adjacent dependency violations could be explained by frontal operculum actively inhibiting representations of both low probability items and short, low probability *n-grams* of contiguous elements (for example, those in which the constituent elements are not suitably ordered).

Salience filtering enables non-adjacent dependencies to be encoded into representations identical to their adjacent counterparts, for any length of dependency fitting into working memory. That is, for any salient **A** and **B** and *n* irrelevant **X** elements, the sequence **A-X***^n^***-B** may be rendered into the same encoding as **AB** alone by selectively inhibiting downstream encodings of irrelevant **X** representations (figure 3, identical top representations, left and middle diagrams). Discarding irrelevant items results in a more efficient representational code, and in state-transition terms allows for grammaticality judgements to be made on *n*th-order non-adjacent dependencies using only a first-order Markov process.

### (c) Hierarchical dependencies

As shown, within our model a dependency comprises multiple superposed *position* ⨂ *item* bindings. Although the dependency can incorporate items retrieved from either contiguous or discontiguous positions in serial working memory, the recoded dependency representation can in both cases be considered a chunk (in figure 3, left diagram, the chunk is **A**-**B**; in the right diagram, the two chunks are **A1**-**B1** and **A2**-**B2**). To encode hierarchical dependencies (figure 3, right diagram, showing nested dependencies), every *dependency* needs to be bound with a unique positional tag, as for individual items. Like individual items, these can also be superposed, forming a superchunk containing a higher-order dependency representation. This process can be recursively repeated to form a single reduced representation of the hierarchical structure of the entire input sequence, integrating progressively increasing amounts of information at higher hierarchical levels of encoding.

The above system is sufficient to compress hierarchical structure into a reduced representation. However, we must encode the reduced representation using more than item positional tags if we are to support unambiguous recovery of specific constituents and comfortably discard the original representations. To avoid generating identical codes for dissimilar structures, we can define sets of positional tags specific to each level of the hierarchy, for example **1**_**I**_° and **2**_**I**_° for the first and second items, **1**_**C**_° and **2**_**C**_° for the first and second chunks, and so on (figure 3, right diagram). For this reason, it can be computationally beneficial to define all positional tags as convolutional powers (i.e. base^exponenent^) of a given *base vector* through repeated self-binding (i.e. 2° = 1°⨂1° = (1°)^2^). This, or a similarly invariant function, can be learned by a network such that tags are encoded as a function of not just position (by varying the exponent), but also context (by varying the base vector). Crucially, by binding items only to a finite set of deterministically generated positional tags, the modelled system can always undertake unbinding by re-instantiating the same set of keys; by iterating through every possible tag, all constituent items can be retrieved in sequence.

A given dependency coding network can retrieve and recode items from arbitrary positions in the linear sequence buffer, provided it has sufficient integrative power. Therefore, this representational scheme is capable of encoding not just nested dependencies, but other types of hierarchical dependency such as crossed dependencies. For example, figure 3 (right diagram) illustrates retrieval of items 1 and 4 (A1 and B1) from sequence memory, which are chunked through binding and superposition; and retrieval of items 2 and 3 (A2 and B2), which are likewise chunked. These two chunks form a superchunk representing a nested dependency. Cross-serial dependencies between words are notably also present in some human languages. Like nested dependencies, these can also be readily represented by our model. We could, for example, have retrieved and bound items 1 and 3, and 2 and 4, respectively, to form a crossed dependency structure. This is illustrated within our coded demonstration (doi:10.5281/zenodo.3464607). The inclusion of cross-serial dependencies alters the minimum computational requirements for any parsing agent [110], and thus it is important that a domain-general representational model has the potential to account for them as well as other language-like hierarchical constructions.

Having recoded a linear sequence in terms of its dependencies, it is possible to recover one or more specific items from the representation of hierarchical structure by unbinding using keys that specify context-specific position(s). With reference to figure 3 and using this method: the key **¬1_C_°** will recover all position–item bindings of the first chunk; the key **¬2_I_°** will recover the second item of every chunk (with each still bound to information on the requisite chunk position); and the key **¬(2_I_°** ⨂ **1_C_°)** will recover the second item of the first chunk. This flexible decoding scheme is important because it readily supports manipulation of entire chunks and generalization of dependencies to multiple timescales.

A natural consequence of the above encoding scheme is that the superposition of many dependency representations over time can gradually give rise to a memory trace. This trace will be influenced by the probabilistic distribution of dependencies over the set of input sequences, analogous to implicit learning. It is now possible to computationally model functional characteristics of hippocampal subregions, and by this method, it has been proposed that a monosynaptic (entorhinal cortex to CA1) pathway possesses the relevant properties to support implicit learning [40]. This is relevant for sequence learning, where dependencies are established over many trials. Single-trial learning, by contrast, requires processing by further hippocampal subregions. This suggests a prominent role for parts of the hippocampal system in sequence processing, in line with recent findings [39,40]. However, this is not to say that we should expect activation of the hippocampus to be constant throughout. Indeed, the human neurobiology literature suggests that there is a decrease in hippocampal involvement over time when learning an artificial grammar [111], or acquiring a novel semantically meaningful lexicon [112]. These observations can be interpreted by way of a predictive coding account, in terms of the degree of mismatch between stored and incoming sensory encodings [113]. We can relate this associative mismatch account of hippocampal involvement to the idea of a superposed memory trace. Namely, any newly presented sequence is likely to be more similar to a superposition of encountered sequences (weighted to simulate rehearsal) as time progresses and the number of constituents in the superposition grows. Thus, the degree of mismatch, and any activation requisite for such an encoding, is likely to decrease over time.

We propose that hierarchical structure-building is one of the key roles of the dorsal aspect of ventrolateral prefrontal cortex (dorsal VLPFC, incorporating Brodmann areas 44 and 45). This position is supported by human neurobiological evidence on syntactic processes prominently featuring hierarchical dependencies [34,38]. It has been proposed that at least BA44 supports a recursive, multi-dependency management process, a fact that would explain increases in its activation observed with increasing depths of hierarchical dependency nesting [114]. The recursive reuse of a consistent architecture by VLPFC would be consistent with our recursive use of vector symbolic operations during the encoding of hierarchical dependencies. Repeated superposition of sparse dependency representations will manifest as an increase in local activity as increasing numbers of neurons support the representation; as an example of this effect, consider the increased activity represented at the top of figure 3, relative to the bottom. A ‘reset’ (or re-sparsification) of the buffer will result in a sudden drop in population activity. Such neural accumulation and reset activity has been identified within human intracranial recordings in subjects listening to sentences containing words that accumulate into phrases [28]. Furthermore, representations of constituents might not persist beyond the need to encode the reduced dependency representation, a fact that highlights an interesting property of the model: detectable neural delay activity does not need to persist throughout working memory maintenance of the input sequence, consistent with recently reported findings on the neurobiology of working memory [115].

It must be emphasized that the flow diagrams in figure 3 only show *symbolic* information flow. There are likely to be sub-symbolic influences acting over time to hone the associated neural activation patterns, requiring feedback and feedforward interactions between neural ensembles in the model. These are briefly alluded to in figure 3 as a single exemplar (curved, dashed arrow, **left**) denoting top-down influences acting on sensory cortical representations. These sub-symbolic influences may be investigated further by instantiating a dynamic neural implementation of the model via the NEF [83], where learning algorithms employing, for instance, spike-timing-dependent plasticity can be applied to learn functions over time [116].

## 7. In conclusion: predictions emerging from the structure of VS-BIND

Vector symbolic operations implemented in artificial neural networks, as we have established, have the potential to further our understanding of combinatorial binding at neural, cognitive and behavioural levels, generating site-specific neural correlates ripe for testing. Testing falsifiable predictions made by this model is important to provide evidence about the plausibility of the combinatorial operators and processes modelled here.

The components of VS-BIND we have outlined here suggest the following predictions:

— *Superposition of sparse vectors* manifests as steadily increasing net activation [28].
— *Binding produces orthogonal vectors* by re-coding, which might be observed as reductions in neural responses, for example, reductions in high gamma activity during neural local field potential recordings [28].
— *Chunking operations* are intrinsic to linguistic bracketing and both produce and depend upon dynamic patterns of both increasing and decreasing oscillatory activity [26].
— *Relative ordinal position* is a crucial neural code when binding serial input in artificial and biological neural systems, indicating that time-sensitive or serial-position-sensitive cells, wherever they reside [101], are indispensable for complex combinatorial binding in sequence processing.
— *Hippocampal system involvement* is not only required for recurrent activity to establish associations during Hebbian learning, but components of this system are also involved in structured sequence processing, establishing rules and dependencies across temporal scales through interactions with at least inferior frontal cortex.

In summary, the VS-BIND model describes how sequences can be processed and represented using established VSA representations and operations, with the main goal of generating falsifiable predictions of neural correlates. While it was beyond the scope of this paper to model the learning processes, VSA-based models such as ours could now be extended with spike-timing-dependent plasticity or other learning and memory algorithms.

It is also important to note that, although our model can represent novel sequences of arbitrary items, the model does not bind arbitrary items together, which risks producing uninterrogable or unrecoverable bound representations. Instead, VS-BIND only uses the binding operator to combine an item with a known positional or contextual tag. Critically, these positional tags are known to the model, being drawn from a small, stable set of codes that are deterministically re-instantiated by dlPFC neurons or hippocampal ‘time cells’. This process allows retrieval of arbitrary items without the need for modeller-specific knowledge or a look up table: all that is needed is the set of positional tags and the sequence representation. Such models could, in the future, be combined with systems that explicitly support the binding together of arbitrary items, for example, to flexibly manipulate linguistic relations in working memory [52].

We have here presented a blueprint for a neurobiologically plausible computational model, VS-BIND, outlining its principal mechanisms for encoding sequence dependencies. We have also highlighted aspects of VS-BIND that support ongoing efforts to simulate cognitive functions relevant to segmentation, chunking, recall and prediction using VSAs within spiking neural networks. Further developments, in conjunction with other models, such as those that are classically compositional approaches aimed at modelling language-specific or linguistic properties, carry tremendous potential to better understand fundamental aspects of cognition and to guide the pursuit of neurobiological correlates of complex mental structures.

## Data accessibility

Supporting Matlab code can be found at doi:10.5281/zenodo.3464607 [23].
